# Oncofusions – shaping cancer care

**DOI:** 10.1093/oncolo/oyae126

**Published:** 2024-06-04

**Authors:** Giovanna Dashi, Markku Varjosalo

**Affiliations:** Institute of Biotechnology, HiLIFE Helsinki Institute of Life Science, University of Helsinki, Helsinki, Finland; iCAN Digital Precision Cancer Medicine Flagship, University of Helsinki, Helsinki, Finland; Institute of Biotechnology, HiLIFE Helsinki Institute of Life Science, University of Helsinki, Helsinki, Finland; iCAN Digital Precision Cancer Medicine Flagship, University of Helsinki, Helsinki, Finland

## Abstract

Cancer manifests through a spectrum of mutations, including gene fusions termed oncofusions. These structural alterations influence tumorigenesis across various cancer types. Oncofusions arise primarily from genomic rearrangements and operate through deregulation or hybrid gene formation mechanisms. Notable examples such as *BCR::ABL* and *EWS::FLI1* underscore their clinical significance. Several case studies exemplify the role of identifying and targeting oncofusions in guiding treatment decisions and improving patient outcomes. However, challenges persist in discerning drivers from passenger mutations and addressing acquired resistance. Despite advancements, the complexity of oncofusions warrants further exploration of their full potential as therapeutic targets, requiring a multidisciplinary approach integrating genomics, functional studies, and innovative drug discovery strategies to achieve precision in medicine.

## Teaser

The complexity of oncofusions warrants further exploration of their full potential as therapeutic targets. This commentary describes case studies that exemplify the role of identifying and targeting oncofusions in guiding treatment decisions and improving patient outcomes.

## From gene fusions to oncogenic drivers

**Figure 1.**
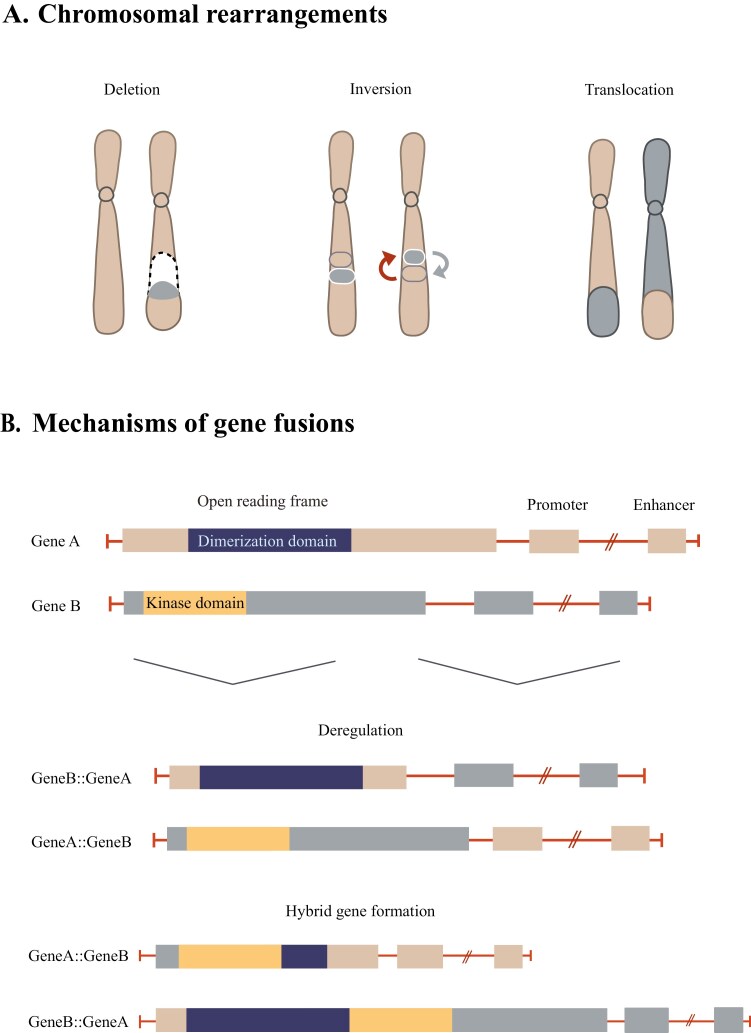
Formation and mechanisms of gene fusions.

Cancer stems from genomic instability leading to wide-ranging genetic changes, from smaller point mutations to sizable gene fusions. Cancer-associated gene fusions or oncofusions account for up to 20% of cancer morbidity and are known drivers of many different cancer types.^1^ Examination of transcriptomic and genomic data has shown that approximately two-thirds of fusion mutations originate from genomic rearrangements, such as deletions, inversions, and translocations.^2^ Oncofusions can function via 2 different mechanisms: deregulation and hybrid gene formation (Figure 1). Deregulation occurs when a gene is fused with a stronger promotor/enhancer of a different gene eg, *IGH::MYC* in Burkitt lymphoma. In this context, a gene that has tightly controlled expression once fused to a stronger promoter becomes aberrantly overexpressed, leading to cancer initiation and progression. Hybrid genes form when 2 coding regions of genes fuse to form an in-frame sequence that results in a fully functional chimeric protein. Examples of well-known hybrid gene fusions include *BCR::ABL* identified in chronic myeloid leukemia, *EWS::FLI1* in Ewing sarcoma, and *EML4::ALK* in lung cancers. Through 5 case studies, the current issue of *The Oncologist* will emphasize the clinical importance of identifying and targeting hybrid gene fusions and how they are useful for precision oncology.

Hybrid gene fusion research emerged in the 1960s with Nowell and Hungefordt’s discovery of the Philadelphia chromosome (Ph1) in leukemia bone marrow cells, a landmark finding that persists as a diagnostic marker today.^3^ Two decades later Stam et al^4^ pinpointed that Ph1 occurs from a translocation between chromosomes 9 and 22, resulting in the *BCR::ABL* fusion. Concurrently, Naldini et al^5^ found that ABL was constitutively activated in Ph1+ cell lines, shedding light on the fusion’s functional significance. Structurally, ABL’s N-terminal domain is exchanged with BCR’s coiled-coil domain, wherein the coiled-coil drives ABL kinase oligomerization and renders it constitutively active.^6^ The Catalogue Of Somatic Mutations In Cancer (COSMIC), a carefully compiled mutation database, covers genes fusions known to be involved in cancer, as defined in the Cancer Gene Census. The gene information was curated by an expert literature search supplemented with NGS information from different genetic sources. Drawing from COSMIC data, after the discovery of *BCR::ABL,* there have only been records of novel translocations,^7^ potential gene fusions,^8^ or partly identified gene fusions such as Topoisomerase-tyrosine kinase fusion^9^ (later identified as *TMP3::NTRK1*), or *RET* fusion with an unknown gene^10^(later identified as *CCDC6::RET*). The second recurring oncofusion to be referred to as a pair of 2 genes is *TCF3::PBX1*, followed by *PML::RAR* and *RUNX1::RUNX1T1* identified in different hematological malignancies^11,12^ and *EWS::FLI1* identified in Ewing sarcoma.^13^ Methods available at the time increased fusion identification; however, it was the advent of NGS that significantly expanded their detection in different cancer types.^14^ These genetic changes alter protein structure, interactions, localization, expression, and stability—causing changes in critical cellular processes such as proliferation, differentiation, and survival.^15^ Subsequent computational analysis of the available sequencing data demonstrated that oncofusions are prone to involve known oncogenes, kinases, chromatin-modifying proteins, and transcription factors.^16-18^ The COSMIC database shows that only 10 out of 305 oncofusions have been extensively documented, these include the aforementioned driver fusions discovered in the late 1900s, indicating that the majority of oncofusions are significantly lacking functional investigation.

The discovery of BCR::ABL and the kinase inhibitor imatinib revolutionized cancer care and research, marking the transition toward precision medicine. Yet, it was the widespread integration of NGS in clinical settings that truly advanced precision oncology. It is now common practice to acquire a comprehensive cancer molecular profile as a continuous guide to therapy decisions throughout treatment. Complete molecular profiling includes tests commonly used in clinics such as NGS panels, whole-genome, and exome sequencing with the addition of proteomics, transcriptomics, high-throughput drug sensitivity testing, and many more to understand the underpinnings of a specific tumor. Groups of expert physicians, scientists, and health care providers join together in what are collectively referred to as molecular tumor boards (MTBs) to analyze such complex information and decide on treatment options and management. These compiled approaches have shifted conventional cancer care and drastically improved precision oncology.^19^ As a result, the National Comprehensive Cancer Network issues guidelines for mutation testing in recurrent oncogenes *EGFR*, *ALK*, *ROS1*, *BRAF*, and *NTRK* intending to identify rare mutations for which effective drugs may be available. The same strategy can be applied to receptor tyrosine kinase (RTK)-oncofusions for which many approved first, second, and third-generation kinase inhibitors are available (Table 1).^21^

**Table 1.** FDA-approved tyrosine kinase inhibitors and their drug targets

Name	First approval	Targets
Imatinib	2001	KIT, RET, NTRK1, CSF1R, PDGFRA, DDR1, ABL1, PDGFRB
Gefitinib	2003	EGFR
Erlotinib	2004	EGFR, NR1I2
Sorafenib	2005	BRAF, RAF1, FLT4, KDR, FLT3, PDGFRB, KIT, FGFR1, RET, FLT1
Dasatinib	2006	ABL1, SRC, EPHA2, LCK, YES1, KIT, PDGFRB, ABL2, FYN
Sunitinib	2006	PDGFRB, FLT1, KIT, KDR, FLT4, FLT3, CSF1R, PDGFRA
Lapatinib	2007	EGFR, ERBB2
Nilotinib	2007	ABL1, KIT
Pazopanib	2009	FLT1, KDR, FLT4, PDGFRA, PDGFRB, KIT, FGFR3, ITK, FGFR1
Crizotinib	2011	ALK, MET
Axitinib	2012	FLT1, KDR, FLT4
Bosutinib	2012	BCR, ABL1, LYN, HCK, SRC, CDK2, MAP2K1, MAP2K2, MAP3K2, CAMK2G
Cabozantinib	2012	MET, KDR, RET
Ponatinib	2012	ABL1, BCR, KIT, RET, TEK, FLT3, FGFR1, FGFR2, FGFR3, FGFR4, LCK, SRC, LYN, KDR, PDGFRA
Regorafenib	2012	FLT1, KDR, FLT4, KIT, PDGFRA, PDGFRB, FGFR1, FGFR2, TEK, DDR2, NTRK1, EPHA2, RAF1, BRAF, MAPK11, FRK, ABL1, RET
Afatinib	2013	EGFR, ERBB2, ERBB4
Ibrutinib	2013	BTK
Ceritinib	2014	ALK
Alectinib	2015	ALK
Lenvatinib	2015	FLT1, KDR, FLT4, FGFR1, FGFR2, FGFR3, FGFR4, KIT
Osimertinib	2015	EGFR
Acalabrutinib	2017	BTK
Brigatinib	2017	ALK, EGFR
Neratinib	2017	ERBB2, EGFR
Dacomitinib	2018	EGFR
Gilteritinib	2018	FLT3, AXL, ALK
Larotrectinib	2018	NTRK1, NTRK2, NTRK3
Lorlatinib	2018	ALK, ROS1
Entrectinib	2019	NTRK1, NTRK2, NTRK3, ROS1, ALK
Erdafitinib	2019	FGFR1
Pexidartinib	2019	FLT3, KIT, CSF1R
Capmatinib	2020	MET
Tucatinib	2020	ERBB2
Infigratinib	2021	FGFR1, FGFR2, FGFR3
Tepotinib	2021	MET
Tivozanib	2021	FLT1, KDR, FLT4
Futibatinib	2022	FGFR1, FGFR2, FGFR3, FGFR4
Fruquintinib	2023	FLT1, KDR, FLT4
Quizartinib	2023	FLT3, CSF1R, KIT

We used the PKIDB open-access data repository (https://www.icoa.fr/pkidb/) as a resource and included all tyrosine targeting inhibitors with a cancer-related indication.^20^

## Oncofusions in oncology today

Large-scale sequencing initiatives like The Cancer Genome Atlas (TCGA), coupled with the ongoing integration of NGS into standard clinical practice, have instigated the identification of gene fusions. These advances pose a question of whether identification is enough, or a more in-depth understanding of their oncogenic potential is necessary.

Analysis of NGS has played a key role in discovering the oncofusion landscape through the characterization of a vast number of cancer samples. In a recent study by Salokas et al (2020), a gene fusion analysis done on a TCGA RNA sequencing dataset identified over 20 000 unique oncofusions of which 29% were predicted to produce in-frame fusion proteins. The most common gene families involving oncofusions are protein kinases and transcription factors. The cancers presented through the 5 case studies discussed in this commentary are all RTK oncofusion positive. Large-scale sequencing results show that out of all in-frame kinase fusions, over 20% fall into RTKs. RTKs are integral membrane receptors driving a variety of intracellular signaling pathways in response to extracellular stimuli. Extensive mutations such as gene fusion can cause drastic changes to RTK phenotypes leading to wide-ranging intracellular abnormalities making them potent oncogenes.

According to COSMIC, 25% of recurring fusions are RTK oncofusions such as *EML4::ALK* and *CCDC6::RET* which have been extensively studied as drivers of many different cancer types (Table 2). Nevertheless, genomic approaches uncovered that a considerable fraction of low-frequency oncofusions have functional effects, opening up the potential for their exploration as actionable targets and novel cancer drivers.^23^ The first case study by Lee et al^24^ presents a 35-year-old patient diagnosed with histiocytosis, a rare multisystem disorder with a phenotype of Erdheim-Chester disease. Due to a lack of common BRAF and MAPK pathway mutations and the limited efficacy of initial therapy, the patient was given palliative radiotherapy to treat the pain. Sequencing analysis revealed an *ALK::TFG* fusion leading the MTB to include the patient in a clinical trial of the ALK inhibitor alectibin. The patient achieved a complete response within a year and continued so for 20 months since therapy initiation. Even though *ALK::TFG* fusions are rare events in histiocytosis, their identification and targeted ALK inhibitor therapy dramatically improved the patient’s quality of life.

**Table 2.** Recurrent oncofusion and the histological subtypes in which they were most frequently identified according to the COSMIC database

BCR::ABL1 (5483)
acute_leukaemia_of_ambiguous_lineage
acute_lymphoblastic_B_cell_leukaemia
acute_lymphoblastic_leukaemia
TMPRSS2::ERG (2639)
adenocarcinoma
glandular_intraepithelial_neoplasia_Grade_III
small_cell_carcinoma
EWSR1::FLI1 (1333)
alveolar
embryonal
giant_cell_tumour
PML::RARA (725)
acute_myeloid_leukaemia
acute_myeloid_leukaemia_therapy_related
chronic_myelomonocytic_leukaemia_therapy_related
EML4::ALK (720)
adenocarcinoma
non_small_cell_carcinoma
squamous_cell_carcinoma
KIAA1549::BRAF (613)
astrocytoma_Grade_I
astrocytoma_Grade_II
astrocytoma_Grade_IV
CCDC6::RET (593)
adenocarcinoma
non_small_cell_carcinoma
papillary_carcinoma
SS18::SSX1 (577)
biphasic
monophasic
poorly_differentiated
RUNX1::RUNX1T1 (417)
acute_lymphoblastic_leukaemia
acute_myeloid_leukaemia
acute_myeloid_leukaemia_therapy_related
PAX3::FOXO1 (382)
alveolar
embryonal
mixed
NCOA4::RET (358)
follicular_lesion-atypia_of_undetermined_significance
Hashimotos_thyroiditis
papillary_carcinoma
ETV6::RUNX1 (357)
acute_lymphoblastic_B_cell_leukaemia
acute_lymphoblastic_leukaemia
acute_lymphoblastic_T_cell_leukaemia
FUS::DDIT3 (351)
mixed
myxofibrosarcoma
myxoid-round_cell
SS18::SSX2 (348)
biphasic
monophasic
poorly_differentiated
NPM1::ALK (319)
adenocarcinoma
anaplastic_large_cell_lymphoma
Hodgkin_lymphoma
KMT2A::AFF1 (308)
acute_lymphoblastic_B_cell_leukaemia
acute_lymphoblastic_leukaemia
acute_lymphoblastic_T_cell_leukaemia
TCF3::PBX1 (301)
acute_lymphoblastic_B_cell_leukaemia
acute_lymphoblastic_leukaemia
acute_lymphoblastic_T_cell_leukaemia
STIL::TAL1 (290)
acute_lymphoblastic_B_cell_leukaemia
acute_lymphoblastic_T_cell_leukaemia
cutaneous_T_cell_lymphoma
COL1A1::PDGFB (262)
fibrosarcomatous
giant_cell_fibroblastoma
pleomorphic
CRTC1::MAML2 (253)
adenoid_cystic_carcinoma
mucoepidermoid_carcinoma
pleomorphic

The numbers in the brackets represent the number of mutated samples the fusion was identified in. The inclusion criteria for recurrent oncofusions was if they were identified in more than 250 samples.^22^

Depending on the type and stage of cancer, different treatment strategies have to be established, whether chemotherapy, immunotherapy, or targeted therapy. In squamous cell lung cancers (SQCLCs), ICIs are the current recommendation for primary therapy, either as monotherapy or in combination with chemotherapy.^25^ In the second case study by Fang et al^26^, sequence analysis of a tumor from a 70-year-old patient diagnosed with stage IV SQCLC revealed a rare *TFG::MET* oncofusion. Following an extensive MTB discussion, instead of a standard first-line ICI treatment, MET inhibitor crizotinib was administered to the patient leading to a complete loss of the primary lung tumor and partial regression of lymph node metastases. In this example, a rare *TFG::MET* mutation in advanced stages of the disease was successfully targeted at the first line of treatment leading to partial remission for over a year after crizotinib was administered.

A notable shift in 21st-century oncologic care is best seen in the 2017 survey showing how 75% of oncologists in the United States rely on NGS to guide cancer treatment.^27^ A metadata analysis showed that patients with matched targeted therapy had longer progression-free survival and in turn lived longer,^28^ indicating the importance of guided therapy and usage of NGS in combination with other omics throughout patient care. The third case study by Wan et al^29^ showed how oncofusion identification in progressive disease can change the clinical outcome. In this study, a 59-year-old man with intrahepatic cholangiocarcinoma was admitted to the hospital due to high CA19-9 levels (7137 U/mL); the patient was operated and chemotherapy was administered. Subsequently, liver metastases were observed. A 639-cancer-related NGS panel analysis of postoperative tissue uncovered an *RBPMS::MET* oncofusion. Since *MET* fusions have been previously targeted in intrahepatic cholangiocarcinoma case studies, the MTB decision was to start the patient on MET inhibitor crizotinib. One month later, the patient’s liver nodules diminished, and CA19-9 levels decreased leading to partial recovery as a result of targeted therapy.

Cancer development is an evolutionary process marked by “driver” and “passenger” somatic mutations and subclonal selection, driving the progression of the disease. The major difference between the 2 types of mutations is that drivers confer growth advantages while passengers do not.^30^ Prior research has highlighted the correlation between genomic instability and gene fusions, suggesting that nonrecurrent, single-gene fusions could potentially represent passenger mutations.^17^ However, a passenger mutation can become triggered and enter the latent driver classification.^30^ Latent drivers can aid oncogenic transformation, drive metastasis, and cause drug resistance. On its own, a latent driver would not have any function, but in the presence of a driver, or a complicated mutational burden of cancer, it can contribute to a considerable number of cooperative mutational combinations to aid disease progression and resistance.^30^

The distinction between oncogenic drivers and instability-induced passengers among gene fusions remains unclear as seen in the fourth case study by Alves de Souza Costa et al^31^. In this case, a 56-year-old nonsmoker female patient with metastatic non-small cell lung cancer (NSCLC) was admitted for treatment after disease progression. In 2019, the patient was admitted for treatment and sequencing results detected a rare fusion *CD47::MET* initially classified as a variant of unknown significance and the patient was treated for 3 years with nontargeted therapies. After progression, they were admitted again, and resequencing detected *CD47::MET* fusion, and in-depth analysis showed that the *MET* kinase was complete which led the experts to classify the fusion this time as likely oncogenic. Subsequent MET inhibitor capmatinib treatment as a fourth-line therapy led to a complete metabolic response in all sites of active disease.

Oncofusions often emerge as mechanisms of drug resistance, disrupting drug binding sites, altering protein conformations, and initiating aberrant signaling pathways.^32-36^ They are defined by their fusion structure and the complex tumor microenvironment and even though they may share the same fusion partner such as RET in the examples below; it does not indicate that functionally they are similar, nor that RET inhibition will be an equally effective treatment option.^37^ The final case study by McKinley et al^38^ presents a 54-year-old Caucasian male with poorly differentiated pancreatic neuroendocrine carcinoma. Sequencing analysis of liver and pancreatic biopsies and circulating tumor DNA showed an occurrence of *NCOA4::RET* in addition to other mutations. The RET inhibitor selpercatinib was chosen for maintenance therapy. However, a phase 2 clinical trial of selpercatinib involving the patient revealed that his cancer was selpercatinib-resistant leading to disease progression with increased lesions and additional metastasis. Subsequent chemotherapy was ineffective and further progression led to the patient’s death. *RET* fusions were investigated in a recent study of 41 patients with NSCLC. Among patients resistant to EGFR treatment, a subset tested positive for *CCDC6::RET* fusion, enabling successful therapy through combination with a RET inhibitor.^39^ In essence, while oncofusions represent potential therapeutic targets, their response to targeted inhibition can vary significantly, as demonstrated by the *NCOA4/CCDC6::RET* fusion emphasizing the critical need for continued research to understand their role in cancer.

## Oncofusions’ role in the future of precision medicine

These 5 case studies underline the importance of detecting oncofusions to guide patient care. Nevertheless, if true precision is to be acquired, only detecting, and targeting oncofusions (as well as other types of mutations) may not be enough. Cancer is an ever-changing disease that warrants continuous interrogation of the complete tumor environment to best predict how a tumor is going to behave and respond to therapy.

Oncofusions are distinctive targets because they are cancer cell-specific regulators of all tumor processes from initiation, progression, and maintenance to metastasis. Many RTK oncofusions are being detected continuously because they can be readily targeted with available FDA-approved kinase inhibitors. Nevertheless, the majority of hybrid fusions can be made of non-RTK kinases as well as other genes encoding for example transcription factors or chromatin modifiers, and these are being identified at an increased pace.^18,40^ Additionally, oncofusions do not have to necessarily retain the binding pockets of partnering proteins, they can lose them completely or partially turning them into undruggable targets. Even RTK oncofusions with existing inhibitors eventually acquire resistance, taking patient care back to square one and to the oldest treatment option—chemotherapy. Given these limitations, exploration of the unique attributes of oncofusions in cancer drug development is imperative.

The nature of oncofusions being a fusion of 2 genes makes them multifaceted drug targets that can enhance current treatment options. Targeting a previously untargeted part of a fusion can provide a means to surpass resistance. For example, the recent development of the Transforming Acid Coiled-Coil 3(TACC3) inhibitor BO-264 significantly decreased the growth of cells harboring a potent FGFR3::TACC3 hybrid protein.^41^ In addition, by changing their localization oncofusions obtain different interacting partners and these novel interactions disrupt their physiological signaling pathways. It has been observed with BCR::ABL that ABL physiologically shuffles between the nucleus and the cytoplasm, but in the fusion form, it’s trapped in the cytoplasm where it finds most of the interactions.^15^ A study of pediatric mesothelioma with a STRN::ALK oncofusions tested drug sensitivity to 527 oncology drugs, although an obvious sensitivity for ALK inhibitors was detected, other sensitive drugs were found to target ALK downstream partners.^42^ Finally, oncofusions can have diverse partners leading to different isoforms of the same partner pair, as observed in ETV6::NTRK3.^43^ Depending on the extent of the additional gene, the resulting protein can be present in many different conformational states which can hinder drug binding or introduce a different target site.^44^ These features can be well used for future drug discovery, especially in the era of new chemical modalities including protein degraders, molecular glues, antibody-drug conjugates, and gene therapy.

Applying multidisciplinary approaches, combining NGS with other omics, using MTB, and establishing a comprehensive molecular tumor profile is the future of precision medicine. When faced with the question of oncofusions, despite the progress in recent years, increased oncofusion identification calls for in-depth investigation of their role in oncogenesis. It may not suffice to only identify and target an oncofusion, we should rather strive to comprehend their genomic and functional profiles for improved and effective, treatment strategies.
